# Surgery

**Published:** 1898-12

**Authors:** James Swain


### SURGERY.

**Intraperitoneal drainage** after operations may generally be regarded as a failure on the part of the surgeon to satisfactorily complete his work. The terrific mortality after the early ovariotomies—before the part played by bacterial invasion was recognised—is doubtless responsible for the habit of endeavouring to rid the abdominal cavity of decomposable fluids by means of drainage; and the habit being established many find it difficult to discard it, and fail to appreciate the enormous power of absorption of the peritoneum, which is said by Wegner to be capable of taking up 3 to 8 per cent. of the body weight in one hour.

In support of this erroneous principle of drainage many methods have been advocated by their various inventors; but drainage is becoming more and more unnecessary with improved methods, and the cases in which it is really required at the present time are very few. This is fully borne out by Dr. J. G. Clark[1] in a critical review of seventeen hundred cases of abdominal section, which goes to prove "that not only is drainage valueless in the great majority of cases in which it has hitherto been used, and is still used by some surgeons and gynecologists, but that it is frequently productive of harm."

That the drainage-tube may be harmful is shown by the observations of Robb and Ghriskey,[2] who found that in spite of the most careful technique 44 per cent. of the cases in which tubes were used showed some form of organism, and they therefore concluded that some of the deaths from sepsis were distinctly due to the use of drainage.

Clark's objections to intraperitoneal drainage may be thus summed up: 1. There is a traumatic and chemical irritation produced by the drainage material. The long contact of a foreign body alone, and still more if such substance is impregnated with any germicidal chemical, produces a destruction of the endothelium of the peritoneum, and thereby reduces its power of absorption. 2. Healing is retarded. This is almost a necessary corollary, but his tables clearly show that the percentage line of local suppuration practically coincides with the rise and fall of the use of the drainage-tube; and the fact that the organisms found in these cases are mostly those which are normally found in the skin goes far to show that they have gained admission by means of the drain. 3. Drainage is not effective in removing fluids and infectious matter. This has been demonstrated by numerous *post-mortem* examinations. The most that the drain can do after the first few hours is to drain a small pocket which has been shut off from the general peritoneal cavity by adhesions. 4. Infection frequently occurs through the drainage track. The observations of Robb and

[1] *Johns Hopkins Hosp. Rep.*, 1898, vii., Nos. 1—2.
[2] *Bull. Johns Hopkins Hosp.*, 1891, ii., 93.

Ghriskey on this point have already been referred to, and the fact has been further corroborated by Clark himself, who obtained cultures of micrococci (chiefly those found in the skin) in every drain employed, although control cultures were made—from the pus in the inflammatory cases, and from the peritoneal secretions in the non-inflammatory cases—at the time of operation and were negative. 5. Post-operative obstruction of the bowel. Although obstinate constipation, tympanites, nausea and vomiting after operations in which drains are employed are due to a constrained position of the intestines around the drain, the presence of actual obstruction is rare. 6. Fæcal fistula. This also is a rare result of drainage, and occurred only once in the series of seventeen hundred cases, and was due in this instance to a pressure necrosis caused by a glass tube on the bowel. 7. Vesical complications. The inflammatory reaction around the drain may cause irritability of bladder, dysuria, or cystitis; adhesions may prevent the normal distension or contraction of the bladder; or a suppurating cavity may rupture into the bladder. 8. Post-operative hernia. It is difficult to estimate the proportion of cases in which this occurs, but Clark thinks it cannot be less than 8 per cent. of cases in which an extensive drain has been employed.

By carefully comparing the results in one hundred similar pelvic inflammatory cases, drained and undrained, Clark shows that in drained cases suppuration of the abdominal wound, persistent vomiting, tympanites, vesical irritation and post-operative pelvic inflammatory deposits are one and all more common than in undrained cases. Under the same conditions it was found that the mortality of the drained cases was 13 per cent. and that of the undrained cases only 6 per cent. The results of the two classes are widely different, and one can scarcely attribute them to the more serious nature of the drained cases, for in the later cases many were not drained which would formerly have been treated by drainage.

In addition to the ordinary means (such as disinfection of hands, isolation of the general peritoneal cavity during operation, &c.) which most surgeons would naturally adopt in the prevention and removal of infection without the employment of drainage, the author advocates other procedures which are open to discussion. These are, irrigation of the peritoneal cavity; the use of saline infusions into the peritoneal cavity followed by postural drainage; and submammary saline infusions.

Many operators would join issue on the advice to thoroughly irrigate the abdomen with normal salt solution "after every operation where *débris* or normal fluids escape into the peritoneal cavity"; but Clark seems to prefer it, and leaves about a litre of the salt solution in the peritoneal cavity before closing the parietal wound, on the principle that foreign matter is more likely to be easily disposed of when in a finely divided state.

**Postural drainage**—by elevating the pelvis after operation—is advocated to prevent fluids stagnating in the dependent parts of the abdominal cavity and so forming "dead spaces," which may furnish favourable culture media for infective organisms, and also to assist the normal intraperitoneal current in the direction of the diaphragm. The best way of carrying this out is to raise the foot of the bed about twenty degrees and to maintain this position for twenty-four or thirty-six hours. The postural method is regarded as a prophylactic measure against post-operative peritonitis, but is useless as a curative measure when peritonitis is established. It should therefore not be employed in cases of purulent peritonitis or in conditions associated with general peritonitis, as for example in some cases of appendicitis.

A limited experience leads the author to agree with the result of the investigations of Bosc[1] and Claisse[2]—that **sub-mammary saline infusions** are beneficial in septic cases. A litre of salt solution is injected daily beneath the mamma until distinct improvement occurs.

As a result of his **review of all classes of drained cases,** the author reduces the conditions in which drainage is indicated to the following: 1. In cases of appendicitis when a secure closure of the stump of the appendix is prevented by the infiltrated condition of the peritoneum and adjacent tissues; or when the appendix has ruptured and caused a local abscess or general peritonitis. 2. In localised collections of pus in the pelvis a drain must be used. The drainage of these cavities, however, through the peritoneum from above is extremely likely to lead to a fatal peritonitis, and they are best treated by incision and drainage through the vagina without opening the peritoneal cavity at all. 3. In suture of the intestine it may be necessary to employ a drain when there is doubt about the security of the suturing. 4. In excision of fistulous tracks leading from the intestine to the abdominal wall, a sterilised gauze drain should be packed down to the sutured areas in the intestine which is prone to break down and re-establish the track. 5. In cases of purulent peritonitis free drainage through lateral openings in the flanks as well as in the centre of the abdomen is frequently necessary.

The list here given is shorter than that which the majority of surgeons might adopt, but there is little doubt that with improved technique it will be still further diminished. I have completely closed the abdomen after the excision of a fistulous track leading from the parietes to the intestine; and have elsewhere[3] referred to cases of purulent peritonitis treated successfully by Finney, by thoroughly mopping the intestines until all pus and fibrinous exudate was cleaned away and the abdomen closed without drainage.

[1] *Presse Méd.*, 1895. [2] *Rev. de Chir.*, 1896, xvi. 686.
[3] *Bristol M.-Chir. J.*, 1897, xv. 336.

The use of the drain will be practically discarded in the near future. The enormous power of absorption of the uninjured peritoneum is becoming rapidly recognised, and with simpler methods based on sounder principles the mortality of abdominal operations will become further lessened.

The question of the best treatment for **simple fracture of the patella** has been exercising the minds of our American *confrères*, and able articles on the subject have been written by Drs. Powers[1] and Stimson.[2] The former has taken considerable pains to collect the opinions of eminent surgical authorities, and he finds that $23\frac{67}{71}$ per cent. are deliberately opposed to operation, $13\frac{17}{71}$ per cent. urge it in all cases where competent surgical skill can be rendered amid suitable surroundings, $56\frac{35}{71}$ per cent. recommend arthrotomy and suture in selected cases (wide diastasis, comminution, &c.), and $5\frac{45}{71}$ per cent. favour the use of Malgaigne's hooks.

In other words, over 70 per cent. favour operation in some cases. But operation is not to be lightly undertaken in all cases; for there is an acknowledged mortality of 1.4 per cent. in published cases, and this can scarcely be regarded as the true death-rate.

Powers thinks it is clear that the procedure has a well-fixed place in surgery, but it should only be performed in healthy individuals of suitable age by competent surgeons, after the dangers and advantages have been fully explained to the patient.

Only those cases should be operated upon in which the separation of the fragments exceeds half an inch, or in which there are extensive lateral tears of the capsule as shown by the joint distension and bulging; and operation should be supplemented by early massage and movement of the joint.

Open arthrotomy is the best form of operation. The soft parts should be sewn together carefully; and this procedure alone is considered sufficient by Stimson, who places two or three catgut sutures in the periosteum along the edge of the fracture, or a single stout catgut or silk suture is passed through the tendon and ligamentum patellæ "so that its two strands lie on the front of the bone." Additional sutures are placed in the torn lateral expansions of the capsule. The risk of accident without prolonged immobilisation in cases so treated is apparent, and most of us would prefer to place a wire suture through the fragments themselves, which considerably shortens the treatment and lessens the risk of "refracture," which seems fairly common in Stimson's method.

Both observers seem agreed that the Dutch massage treatment is best for those cases which are unsuitable for operation. This method was introduced in 1885, by Tilanus of Amster-

[1] *Ann. Surg.*, 1898, xxviii. 67. [2] *Ibid.*, 216.

dam. For the first twenty-four or forty-eight hours an elastic bandage is applied to the affected joint, and the massage is commenced about the second or third day. The thigh and leg are massaged twice daily while the fragments are fixed by an assistant. From eight to fifteen minutes will probably be enough at first, but this may be increased later. Between the *séances* the fragments are fixed with plaster and the limb elevated. Passive motion is commenced early, but should not cause pain. The patient is generally up on crutches by the tenth day, and a fortnight later he discards the crutches and walks with two sticks.

Powers says that in an average case so treated the joint effusion disappears about the end of the third week, and by that time the patient will probably be able to flex the leg to 135°, and by the end of about six weeks will be able to walk quite well. He refers to some cases recorded by Zum Busch treated by the method of Tilanus. The patients got about with a simple splint and stick on the second day, and all returned to work in six weeks with power to lift the leg from a high stool while standing on the opposite foot.

JAMES SWAIN.

